# Bone Marrow-Derived Cells May Not Be the Original Cells for Carcinogen-Induced Mouse Gastrointestinal Carcinomas

**DOI:** 10.1371/journal.pone.0079615

**Published:** 2013-11-19

**Authors:** Chen Yang, Liankun Gu, Dajun Deng

**Affiliations:** Key Laboratory of Carcinogenesis and Translational Research (Ministry of Education), Division of Etiology, Peking University Cancer Hospital & Institute, Haidian District, Beijing, China; National Institutes of Health, United States of America

## Abstract

**Aim:**

It has been reported that bone marrow-derived cells (BMDC) can be original cells of mouse gastric cancers induced by *Helicobacter felis* (*H. felis*) infection. However, it is unknown whether BMDCs are also the original cells of mouse gastrointestinal cancers induced by gastric carcinogens *N*-nitroso-*N*-methylurea (NMU) and *H. felis* infection.

**Methods:**

C57BL/6 recipient mice were initially irradiated with 10Gy X-ray, reconstituted with bone marrow cells from the C57BL/6-Tg (CAG-EGFP) donor mice to label BMDCs with green fluorescence protein (GFP). After 4 weeks of recovery, the bone marrow-transplanted mice were given NMU in drinking water (240 ppm) and subsequently infected with *H. felis* by gavage. Eighty weeks later, all mice were euthanized for pathological examination. The BMDCs expressing GFP were detected in tissues using direct GFP fluorescence confocal microscopy analysis and immunohistochemistry staining (IHC) assays.

**Results:**

Neoplastic lesions were induced by NMU treatment and/or *H. felis* infection at the antrum of the glandular stomach and small intestine. In the direct GFP fluorescence confocal assay, GFP(+) epithelial cell cluster or glands were not observed in these gastrointestinal tumors, however, most GFP(+) BMDCs sporadically located in the tumor stromal tissues. Some of these GFP(+) stromal BMDCs co-expressed the hematopoietic marker CD45 or myofibroblasts markers αSMA and SRF. In the indirect GFP IHC assay, similar results were observed among 11 gastric intraepithelial neoplasia lesions and 2 small intestine tumors.

**Conclusion:**

These results demonstrated that BMDCs might not be the source of gastrointestinal tumor cells induced by NMU and/or *H. felis* infection.

## Introduction

Bone marrow-derived cells (BMDCs) possess a wide range of plasticity and tend to migrate through peripheral organs as a result of inflammation and tissue injury [1], [2]. The differentiation pattern and growth regulation of these cells depend largely on local environmental signals and cues [3]. Chronic gastric inflammation, which develops as a consequence of *Helicobacter pylori* (*H. Pylori*) infection, leads to repetitive injury and repair resulting in the development of gastric adenocarcinoma in human [4], [5]. Studies in mice infected with the related mouse-adapted *Helicobacter* species, *H. felis*, have suggested that adult mouse BMDCs are capable of initiating gastric cancer or contributing to the tumor microenvironment [6], [7].

Although *H. pylori* infection is classified as the first class of carcinogen for human gastric mucosa-associated lymphoid tissue lymphoma (MALT lymphoma) and main cause of chronic gastritis and digestive ulceration, it is not certain whether *H. pylori* infection directly induces human gastric adenocarcinomas. *N*-Nitroso compounds are strong gastric carcinogen for many animals. *N*-Nitroso compounds, deficiency of dietary protein and vitamin C, and high salt intake, as well as *H. pylori* infection, are all risk factors of stomach cancers. *N*-Nitroso-*N*-methylurea (NMU), which can be synthesized in the human and animal stomach [8], [9], is a highly reliable animal gastric chemical carcinogen. Various cancers have been induced by NMU in animal models including rodent gastric carcinoma [10], [11]. In this paper, we investigated whether BMDCs can trans-differentiate into cancer cells in the NMU/*H. felis* induced mouse gastrointestinal cancers.

## Materials and Methods

### Animals

**Figure 1:**
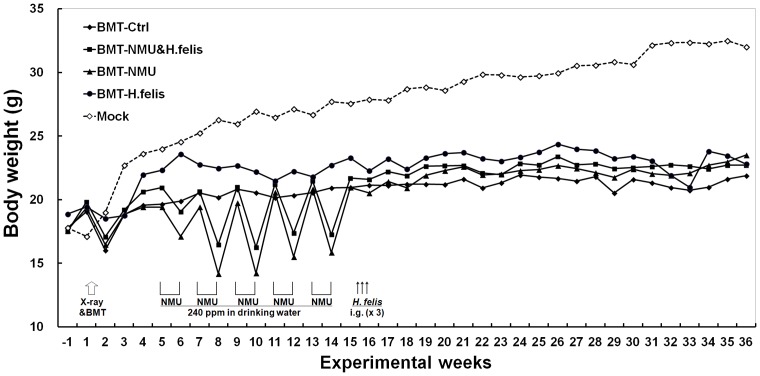
Average body weight of mice with different treatments. BMT, bone-marrow transplantation; Mock, normal mice without any treatment; *H.felis*, gavaged with *H. felis* suspension on three alternate days (one time/day); NMU, drinking water containing 240 ppm NMU in five alternate weeks.

C57BL/6-Tg β-actin-enhanced green fluorescence protein (CAG-EGFP) transgenic mice were purchased from Cyagen Biosciences Inc (Guangzhou). Six weeks old wild type C57BL/6 mice were purchased from Acad. Military Med. Sci. (Beijing) and observed one week before experiments. All mice studies were carried out in a germ-free environment under the approval of Administration Committee on Experiment Animals, Peking University Cancer Hospital and Institute. The body weight of each mouse was recorded every week (Figure 1).

### Bone marrow cell (BMC) isolation

BMCs were flushed from the femurs and tibias of 6–8 weeks old C57BL/6-Tg (CAG-EGFP) transgenic mice. BMCs from the donor mice were pooled together and filtered through a 40 μm cell strainer to produce a single cell suspension in PBS.

### Bone marrow transplantation (BMT)

Seven weeks old recipient C57BL/6 mice (body weight 18–20 g) were irradiated with 10 Gy X-ray, reconstituted with 3**×**10^6^ donor BMCs via a single tail vein injection, and used for experiments after 4 weeks of recovery as described [6]. All recipient mice drank gentamycin-containing water (0.32 g/L) for 14 days immediately following the radiation.

### Administration of carcinogen

The experimental mouse model of gastrointestinal carcinogenesis was setup as described [11]. *N*-Nitroso-*N*-methylurea (NMU, N1517, Sigma, St. Louis, MO) was freshly dissolved in distilled drinking water (concentration, 240 ppm) in light-shielded bottles. Sixty nime BMT mice were divided into 2 groups, 54 mice drank the NMU-containing water on alternate weeks for a total five weeks from the 5^th^ to the 14^th^ experimental week after the BMT. The other 15 mice drank regular drinking water without NMU as negative control.

*H. felis* (ATCC 49179, Rockville, MD) was grown for 48 hours at 37°C under microaerobic conditions on 5% sheep blood agar. 45 NMU-treated mice and 5 drinking-water mice were subsequently infected by intragastric gavage with 1**×**10^8^ colony forming units (CFU) every other day for 3 days (1 time per day) in the 13th experimental week. The remaining 19 mice were gavaged with the broth control.

### Histopathologic examination

Mice were euthanized by CO_2_ inhalation in the 80th experimental week. Their stomachs were opened along the greater curvature. Linear longitudinal sections from the squamocolumnar junction through the pylorus as well as the small intestines were collected. Tissue samples with optically visible neoplasia were frozen with liquid nitrogen and used in direct-fluorescence assay as described below. Most tissue samples from the stomach and intestine were fixed with 10% formalin, embedded in paraffin, cut into 5 μm sections, and stained with hematoxylin-eosin (H&E). Histologic diagnosis was made using the criteria proposed by Leininger and Jokinen [12].

### Immunohistochemistry staining (IHC)

Slides were incubated with primary antibody against GFP (1∶2000; AB290, Abcam, Cambridge, MA) at 4°C overnight. Biotinylated secondary antibody (Zymed, San Francisco, CA) and avidin-biotin DAB detection kit (Zhongshan Jinqiao, Beijing) were used for detection and visualization of GFP(+) cells according to the protocol.

### Direct fluorescence and indirect immuno-fluorescence staining

For GFP direct fluorescence tracing, frozen sections (5 µm) were washed in PBS, counterstained with 1 µg/mL DAPI (Sigma), and viewed with a Leica confocal microscope. After the fluorescence observation, these slides were further H&E-stained.

For dual fluorescence immuno-staining, slides were incubated with primary antibody against CD45 (1∶100; B0848, Anbobio, San Francisco, CA), α-smooth muscle actin (αSMA) (1∶250; AB7817, Abcam) or serum response factor (SRF) (1∶75; AB53147, Abcam) at 4°C overnight, then with TRITC-conjugated secondary antibody (Abcam) for 1 hour at room temperature and observed as described above.

### Flowcytometry analysis

For GFP(+) cell analysis, bone marrow cell suspension was prepared in PBS as described above. Red blood cells were removed by RBC Lysis Buffer (BioLegend, San Diego, CA). The remaining BMCs were analyzed with FACS-LSRII flowcytometer (Becton Dickinson, NJ, USA).

## Results

### Effects of X-ray Irradiation-BMT and Carcinogen Treatments on Mice

To label the BMDCs with GFP, wild type C57BL/6 mice were irradiated with a lethal dose of X-ray (10 Gy) and transplanted with 3**×**10^6^ bone marrow cells derived from C57BL/6-Tg (CAG-EGFP) transgenic mice. Donor cells engraftment was approximately 60% to 90% as assessed by flowcytometry and confocal analysis of GFP(+) cells in the bone marrow suspension and peripheral leukocytes from these recipient mice after 4 weeks' recovery (Figure S1 and S2). These results were similar to the reported engraftment level (60∼80%) [6]. Four control mice without BMT died within two weeks after the radiation, indicating full dysfunction of the bone marrow caused by the radiation. The body weight of BMT-mice in 4 experimental groups was significantly lower than the un-irradiated normal control mice (Figure 1, dash line), indicating long-term effect of the lethal X-ray irradiation and subsequent BMT.

The GFP-chimeric mice were treated with the carcinogen NMU and/or *H. felis* infection and euthanized in the 80^th^ experimental week as described in the method section. The body weight of mice was transiently decreased one week after NMU treatment but was restored to the level for untreated control mice without NMU treatment after one-week interval (Figure 1). No animal died in the BMT-control group (*N* = 10) or the BMT-NMU group (*N* = 9) within 31 experimental weeks, indicating that the general toxicity of the 240ppm NMU treatment was weak and reversible for these BMT-mice. However, 17 mice in the BMT-NMU and *H.felis* group (*N* = 45) and 1 mouse in the BMT-*H.felis* group (*N* = 5) died within 31 experimental weeks after *H. felis* infection. Acute intestine inflammation was observed duing body examination of these mice, indicating the serious intestine toxicity of *H. felis* infection.

**Figure 2:**
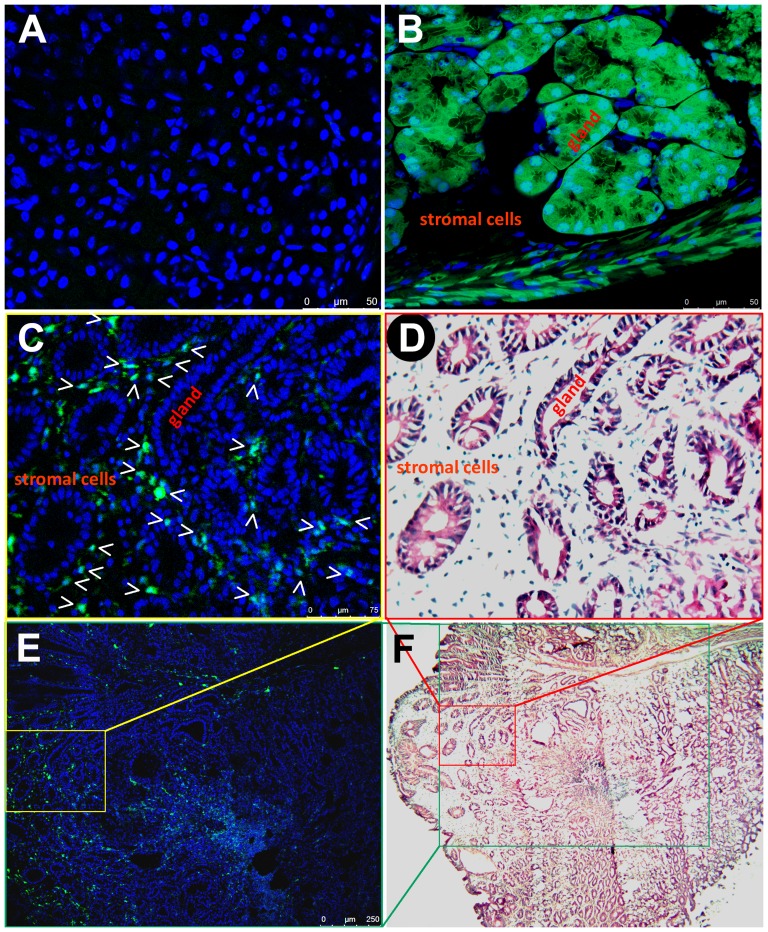
Bone marrow-derived infiltrating cells in the stromal tissue of gastric intraepithelial tumor traced by GFP direct fluorescence. (**A**) Normal tissues of the glandular stomach of a regular GFP(−) control mouse; (**B**) Normal tissues of the glandular stomach of a GFP(+) transgenic control mouse; (**C, E, D, F**) An induced gastric intraepithelial neoplasia (GIN) in a bone marrow transplanted mouse. GFP(+) BMDCs tracked with direct fluorescence localized in the GIN stromal tissue are shown in **C** and **E.** The same GIN lesion slide stained by H&E after the fluorescence observation are shown in **D** and **F**. DAPI (**A**–**C** and **E**) and hematoxylin (**D** and **F**) are used to visualize nuclei, respectively. Locations of the images **C** and **D** in the images **E** and **F**, and the image **E** in the image **F** are marked in the corresponding color. The gastric glands and stromal cells are also labeled.

**Figure 3:**
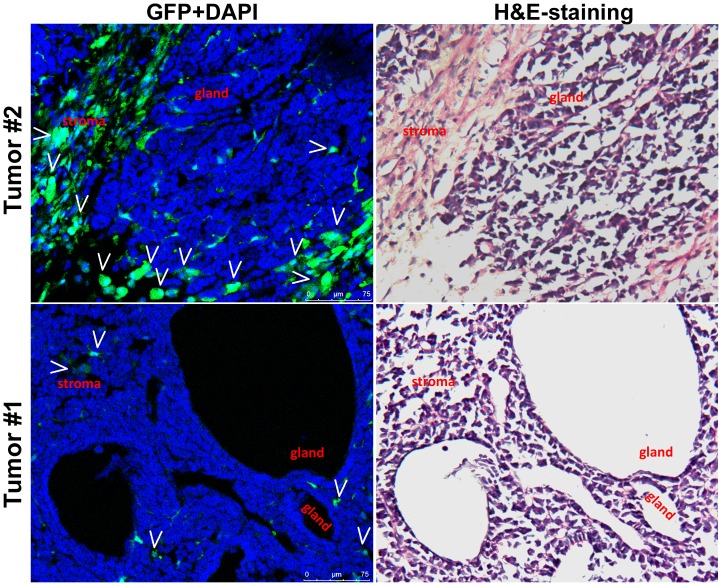
Stromal cells originated from bone marrow-derived cells in two small intestinal tumors. GFP(+) BMDCs indicated by white arrows were observed in the stromal tissues of two small intestinal tumors in the GFP direct-fluorescence assay (left panel). The histological images were further displayed with the H&E staining (right panel). H&E-staining images of paraffin-embedded tissue from the intestinal tumor used in this direct-GFP confocal analysis are displayed in Figure S3.

From the 31^st^ to the 80^th^ experimental week, gastric intraepithelial neoplasia lesions (GIN) of the glandular stomach were pathologically observed in 9 NMU and *H.felis*-group mice and 4 NMU-group mice (Figure 2). Small intestinal tumors were observed in 2 *H.felis*-group mice and 2 NMU and *H.felis*-group mice (Figure 3 and Figure S3). These gastrointestinal tumors were used to investigate whether BMDC cells were the original cells of these tumors.

### Characterization of BMDCs in Carcinogen-Induced Gastrointestinal Tumors

To detect the presence of BMDCs in the carcinogen-treated mouse gastrointestinal tumors, GFP direct fluorescence analysis was performed using the frozen tissue samples with optically-visible neoplasia lesions (one from the glandular stomach and two from the small intestine that were pathologically diagnosed as GIN or small intestine tumors). Frozen sections were stained with DAPI, and viewed under a confocal laser-scanning fluorescence microscope, then further stained with hematoxylin-eosin (H&E). Unlike the previous report [6], the GFP(+) BMDC cell clusters were not observed in the glandular epithelium of GINs (Figure 2), nor in the epithelium of small intestine tumors (Figure 3). Instead, most GFP(+) cells were located in the tumor stromal tissues.

**Figure 4:**
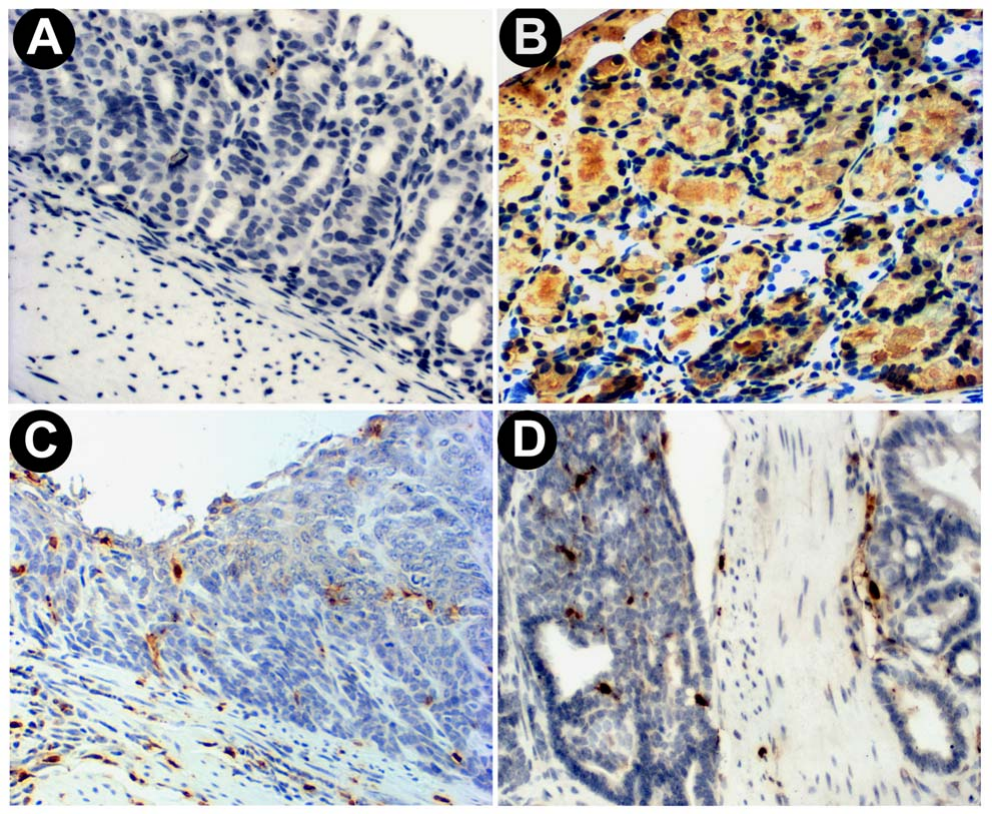
Bone marrow-derived infiltrating cells in the stromal tissue of gastrointestinal tumors displayed with GFP immunohistochemistry staining. (**A**) Normal tissues of the glandular stomach of a regular GFP(−) control mouse; (**B**) Normal tissues of the glandular stomach of a GFP(+) transgenic control mouse; (**C**) An induced gastric intraepithelial neoplasia (GIN) in a bone marrow transplanted mouse; (**D**) An induced small intestinal tumor in a bone marrow transplanted mouse. Hematoxylin was used to visualize nuclei.

To further confirm the results of the direct GFP-fluorescence assay, the indirect GFP-IHC staining assay using an antibody against GFP was used to detect GFP(+) cells in 11 paraffin-embedded GIN and 2 small intestine tumor samples. As expected, most GFP(+) cells were found in the submucosa and stromal tissues around the dysplastic glands (Figure 4C and 4D). No GFP(+) BMDC-derived gland was observed in 11 tested GINs nor in 2 small intestinal tumors (Figure 4, compare 4C and 4D with 4B).

**Figure 5:**
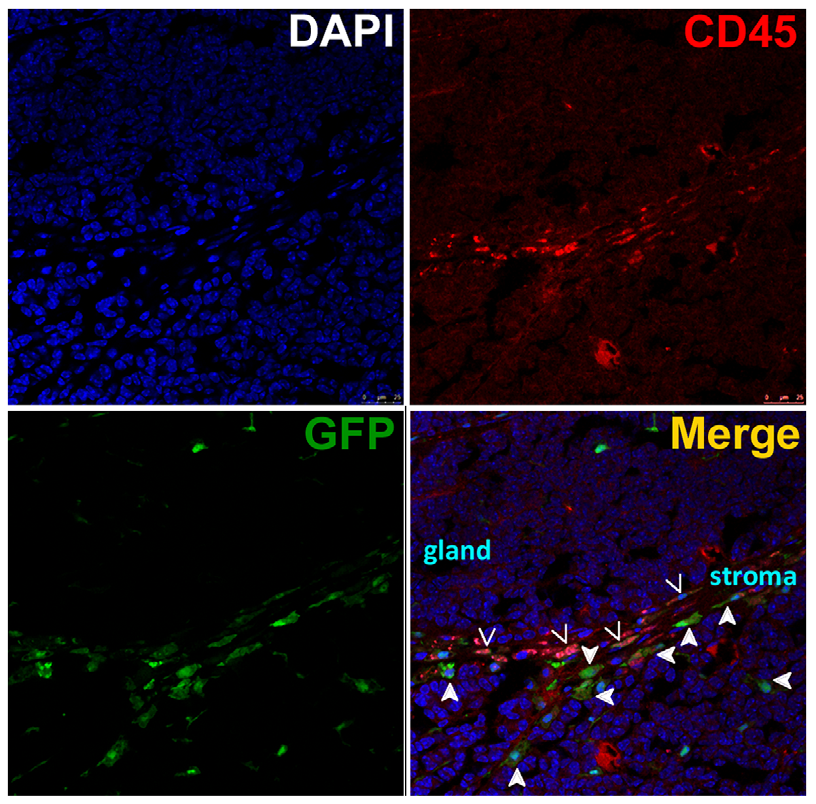
Co-expression of CD45 in part of stromal cells originated from bone marrow-derived cells. The representative images demonstrated that some GFP(+) BMDCs in the tumor stroma co-expressed CD45 (indicated by arrows), a hematopoietic cell marker, in a carcinogen-induced small intestinal tumor. Other GFP(+) cells without CD45 expression are indicated by filled arrows. The intestinal glands and stromal cells are also labeled.

We considered that some GFP(+) stromal cells were likely to be the tumor -infiltrating hematopoietic cells. To investigate the presence of these cells, tissues were stained for CD45, a commonly used marker for hematopoietic cells except erythrocytes and platelets. The results showed that some GFP(+) stromal cells co-expressed CD45 in the intestinal tumor (Figure 5).

**Figure 6:**
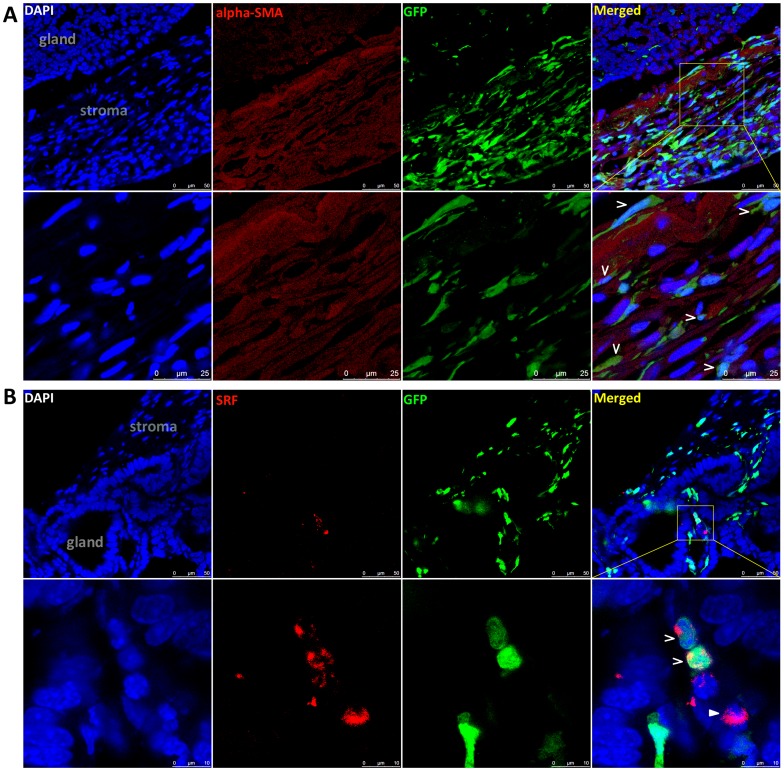
Co-expression of αSMA and SRF in part of stromal cells originated from bone marrow-derived cells. (**A**) Images demonstrating that some GFP(+) BMDCs in the tumor stroma co-expressed αSMA (indicated by arrows) in a carcinogen-induced small intestinal tumor. (**B**) Images demonstrating that some GFP(+) BMDCs in the tumor stroma co-expressed SRF (indicated by arrows) in the small intestinal tumor. A SRF(+) cell without GFP expression is indicated by a filled arrow.

It has been reported that BMDCs can trans-differentiate into myofibroblasts in cancer stroma and promote cancer growth and distant metastasis [7], [13], [14]. To determine whether the GFP(+) stromal cells could be myofibroblasts, the expression status of two myofibroblast-related markers αSMA and SRF in these gastroentestinal tumors were analyzed using cofocal assays. We found that some of the GFP(+) stromal cells indeed co-expressed αSMA and SRF (Figure 6).

## Discussion

It has been reported that BMDCs incorporation occurs in gastrointestinal epithelia associated with elevated proliferation and inflammation [6], [15]. In some mouse models, the recruited BMDCs may contribute to new liver epithelia and prostate regrowth [16], [17]. However, in mouse chronic pancreatitis lesion, the frequency of BMDCs in pancreatic ducts is very low [18]. It has also been reported that BMDCs are not the origin of cancer stem cells in ultraviolet induced skin cancers [19]. In addition, one recent clinical study demonstrated that only a small percentage (approximately 1% to 2%) of the colon neoplastic cells was donor-derived cells after sex-mismatched bone marrow transplantation [20]. In chronic liver fibrosis, BMDCs is recruited to mouse liver tissues as myofibroblasts [21]. In fact, there has been no following report confirming the “bone to cancer” conclusion in the paper published in *Science* [6]. Although the authors used two independent models to label BMDCs, only indirect assays were performed to track the BMDCs in mouse gastric epithelia. False positive observation cannot completely be excluded in experiments using the indirect assays. To avoid false positive results, we used the direct fluorescence assay to trace GFP(+) BMDCs and confirmed the results with indirect IHC. Unexpectedly, we did not observe any GFP(+) glands or GFP(+) epithelial cell clusters in glands in the carcinogen-induced mouse gastrointestinal tumors.

Bone marrow derived-myofibroblasts in cancer stroma may promote mouse gastric cancer growth or lung cancer metastasis [7], [13], [14]. It has also been suggested that BMDCs contributing to the activated pancreatic stellate cell population in chronic pancreatitis and pancreatic cancer have different phenotypes, and may play important roles in these diseases [22]. aSMA is actively expressed in myofibroblasts and used as aregular marker of myofibroblasts [7]. SRF, another protein actively expressed in stromal myofibroblast, is a master regulator of myogenesis and plays a critical role in myofibroblast differentiation [23]–[25]. In the present study, we found that part of GFP(+) stromal cells were also αSMA(+) or SRF(+) myofibroblasts, and some of them were CD45(+) hematopoietic cells in mouse *H. felis*/NMU-induced gastrointestinal tumors. This is consistent with the previous report that BMC cells that migrated to *H. felis*-induced gastric tumors can trans-differentiate to myofibroblasts [7].

Small intestine cancer is a rare cancer in human and its cause is unknown. Interestingly, in the present study, four small intestine cancers were induced by *H. felis* infection in mice with the bone-marrow transplantation. Frequent mouse death and acute intestine inflammation were observed in the BMT-mice (with or without NMU treatment) after *H. felis* infection. The reason may be that these BMT-mice become susceptive to the toxicity and carcinogenicity of *H. felis* infection after the lethal X-irradiation. We found that *p16* methylation was very common in somatic cells in different organs including the stomach and small intestine in these irradiated mice (Yang C, et al. unpublished data). It is worth to evaluate the specific toxicity of *H. pylori* infection among patients with frequent radiotherapy.

In conclusion, the present study suggests that “bone to cancer” trans-differentiation may not be the true source of gastrointestinal tumors, or at least not a general phenomenon. Instead, BMDCs might be involved in cancer development through cancer-stromal cell interaction.

## Supporting Information

Figure S1 **Hematopoietic reconstitution was evaluated in the bone marrow suspension 4 weeks after bone marrow transplantation.** (**A**) Flow-cytometry analysis of the proportion of GFP(+) cells in all nucleated bone marrow cells. Results from 16 BMT mice are displayed; (**B**) GFP direct fluorescence images of a wild type GFP(−) control, transgenic GFP(+) control, and three BMT mice were displayed. DAPI is used to visualize the nuclei (blue). (**C** and **D**) less than 2% of the bone marrow cells from the wild type mouse are GFP-positive, whereas 83.3% of the bone marrow cells from the β-actin-EGFP transgenic mouse are GFP-positive in the flow-cytometry analysis (inserted charts). The merged images for two control mice are displayed as two left images in **B**.
(TIF)

Figure S2 **Demonstration of hematopoietic reconstitution in the peripheral leukocytes in the confocal analysis 4 weeks after bone marrow transplantation.** The peripheral leukocytes from a transgenic GFP(+) mouse and a wildtype GFP(−) mouse are used as positive and negative controls.
(TIF)

Figure S3 **H&E-staining images of paraffin-embedded tissue from the intestinal tumor used in the direct-GFP confocal analysis (** **Figure 3** **,** **5** **, and** **6** **).**
(TIF)
