# Supplementary figures and images for: Bone Marrow-Derived Cells May Not Be the Original Cells for Carcinogen-Induced Mouse Gastrointestinal Carcinomas
Source: PLoS One. 2013 Nov 19;8(11):e79615. doi: 10.1371/journal.pone.0079615 (PMC3834118; doi:10.1371/journal.pone.0079615)

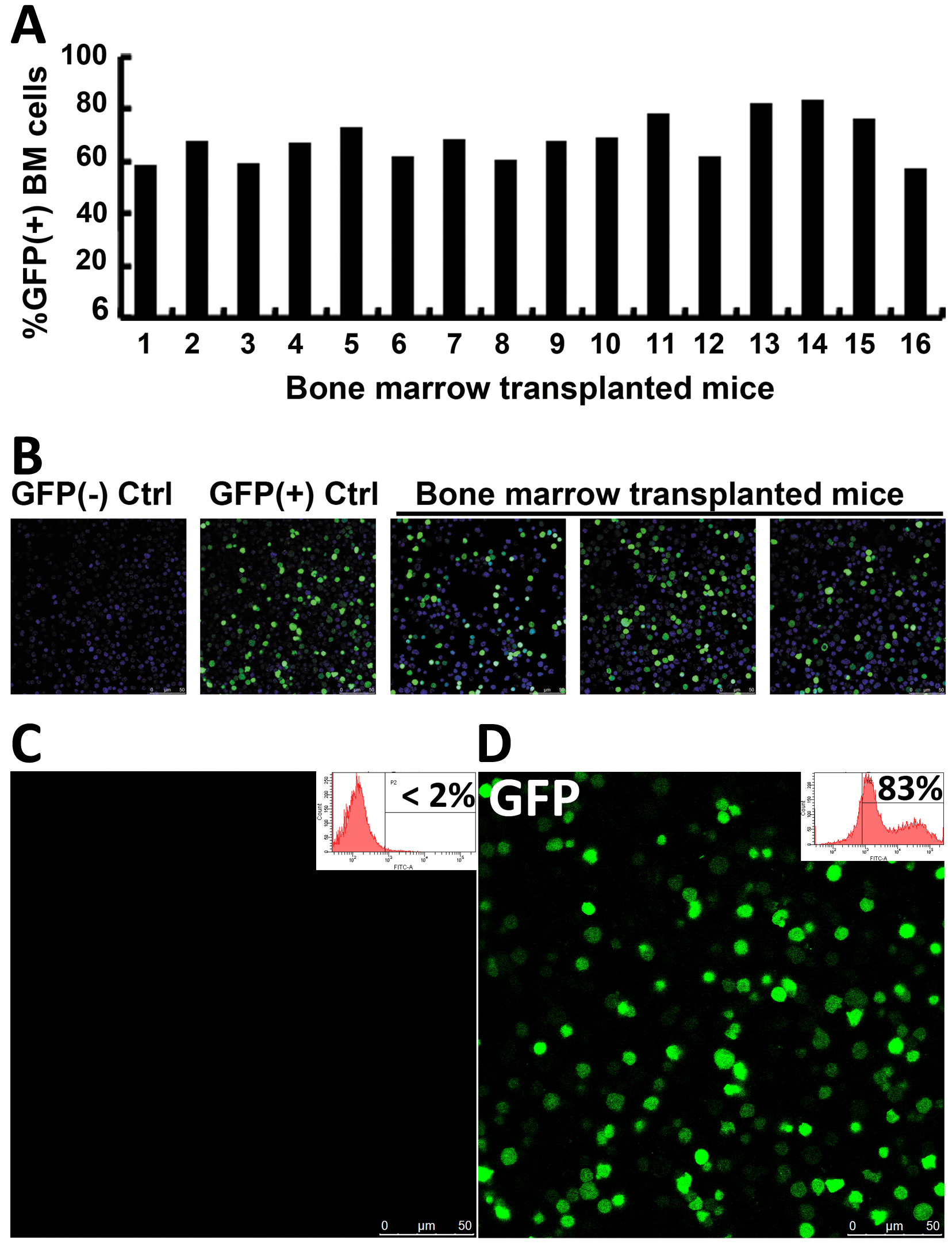

Supplement: Figure S1 — Hematopoietic reconstitution was evaluated in the bone marrow suspension 4 weeks after bone marrow transplantation. (A) Flow-cytometry analysis of the proportion of GFP(+) cells in all nucleated bone marrow cells. Results from 16 BMT mice are displayed; (B) GFP direct fluorescence images of a wild type GFP(−) control, transgenic GFP(+) control, and three BMT mice were displayed. DAPI is used to visualize the nuclei (blue). (C and D) less than 2% of the bone marrow cells from the wild type mouse are GFP-positive, whereas 83.3% of the bone marrow cells from the β-actin-EGFP transgenic mouse are GFP-positive in the flow-cytometry analysis (inserted charts). The merged images for two control mice are displayed as two left images in B. (TIF) [file pone.0079615.s001.tif]

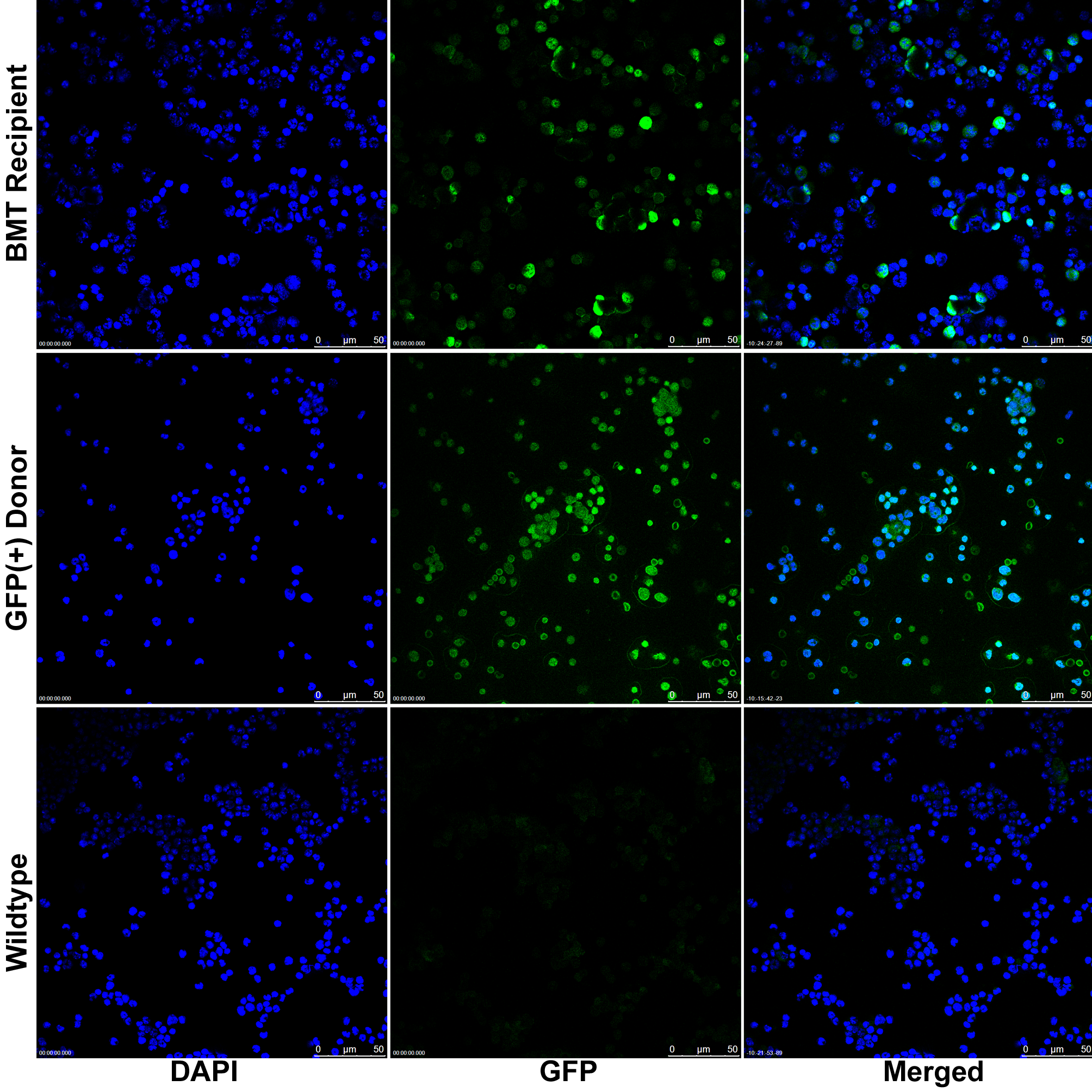

Supplement: Figure S2 — Demonstration of hematopoietic reconstitution in the peripheral leukocytes in the confocal analysis 4 weeks after bone marrow transplantation. The peripheral leukocytes from a transgenic GFP(+) mouse and a wildtype GFP(−) mouse are used as positive and negative controls. (TIF) [file pone.0079615.s002.tif]

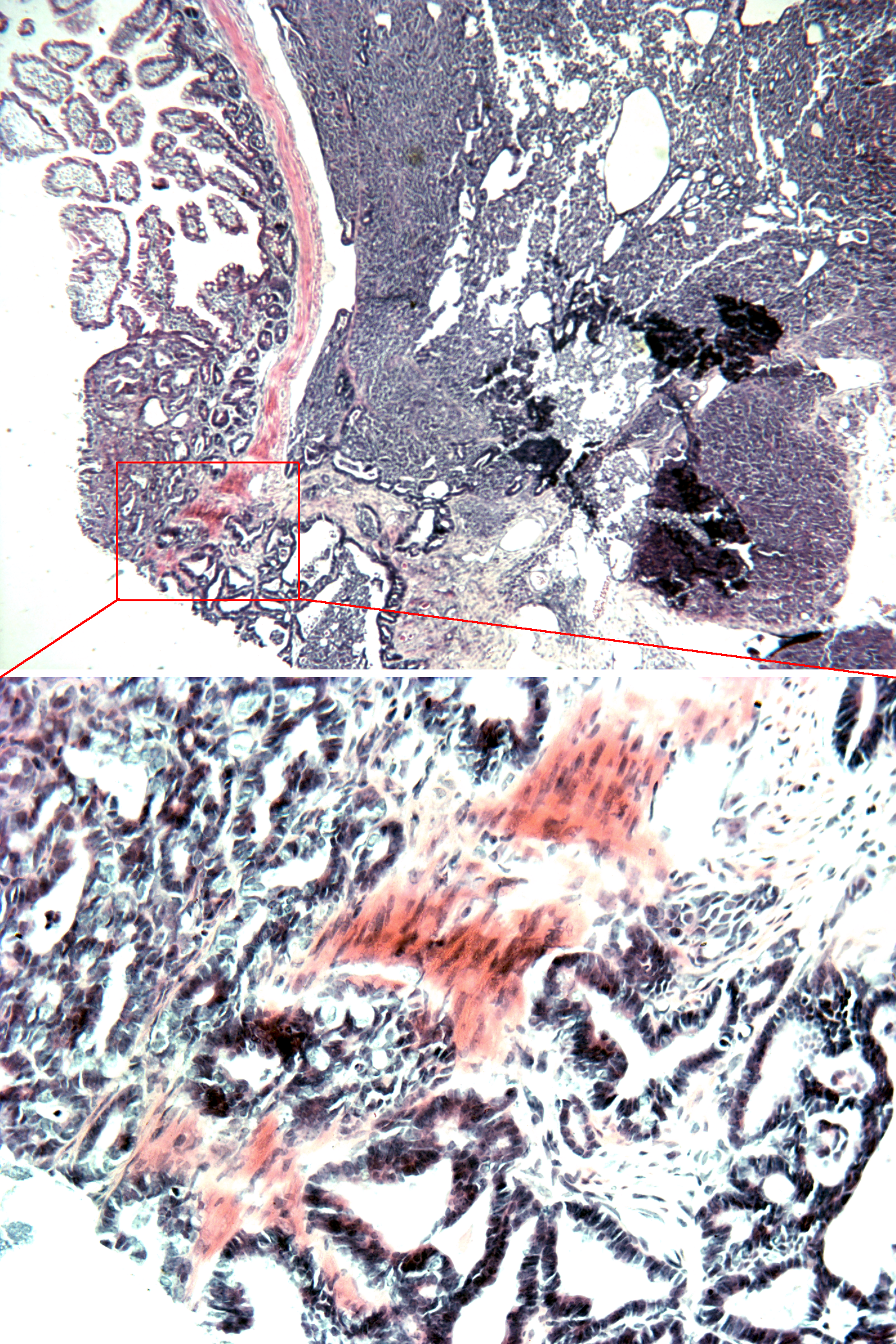

Supplement: Figure S3 — H&E-staining images of paraffin-embedded tissue from the intestinal tumor used in the direct-GFP confocal analysis ( Figure 3 , 5 , and 6 ). (TIF) [file pone.0079615.s003.tif]
